# Comparative pharmacokinetic evaluation and bioequivalence study of three different formulations of Imatinib Mesylate in CML patients

**Published:** 2015-10-01

**Authors:** Ehsan Mohajeri, Behjat Kalantari-Khandani, Abbas Pardakhty, Moeinadin Safavi, Mehdi Ansari

**Affiliations:** 1 Department of Pharmaceutics, Faculty of Pharmacy, Kerman University of Medical Sciences, Kerman, Iran; 2 Department of Oncology and Hematology, Faculty of Medicine, Kerman University of Medical Sciences, Kerman, Iran; 3 Pharmaceutics Research Center, Kerman University of Medical Sciences, Kerman, Iran; 4 Department of Pathology, Afzalipour Medical Faculty, Kerman University of Medical Sciences, Kerman, Iran

**Keywords:** Imatinib, Bioequivalence, pharmacokinetics

## Abstract

**Background:** Imatinib is known as the drug of choice for treatment of chronic myeloid leukemia (CML). For adults the recommended daily dosage of 400 mg requires simultaneous intake of up to four capsules or tablets each 100 mg. A new 400 mg film coated tablet developed due to the need to swallow multiple capsules or tablets per dose and that was a negative impact on treatment adherence.

**Subjects and Methods:** A group of 36 patients were randomly assigned to receive Imatinib as 4×100 mg capsules, 4×100 mg tablets and 1×400 mg tablet. Blood sampling was performed for up to 48 h after first dosing. After that, subjects were monitored to assess drug related adverse events. Pharmacokinetic parameters were assessed using concentration-time curves for plasma Imatinib and its metabolite.

**Results:** Mean area under the curve (AUC_ (0–∞)_) values were 27011, 25811 and 25699 ng/ml for 4×100 mg capsules, 4×100 mg tablets and 1×400 mg tablets, respectively. C_max_ values were 1548, 1605 and 1622 ng/ml and t_1/2 _values were 15.7, 15.8 and 15.6 h. The Test/Reference ratios for AUC_ (0–∞)_, AUC_ (0–48)_, and C_max_ were 0.99, 0.99 and 1.02 for 4×100 mg tablets versus 4×100 mg capsules and 0.96, 0.96 and 0.99 for 1×400 mg tablet versus 4×100 mg capsules. The 95% confidence intervals were fully contained within the accepted interval. The mild and moderate adverse events considered to be drug related were reported. These events showed no clustering by type of dosage form and were of little to no clinical significance.

**Conclusion:** Film coated (400 mg) tablet dosage formulations of Imatinib is bioequivalent to the commercial available 100 mg hard gelatin capsule, and is as safe and well tolerated.

## Introduction

 Imatinib is a tyrosine kinase inhibitor that targets BCR-ABL1, platelet-derived growth factor receptors (PDGFRs) and c-KIT receptors for stem cell factor. Constitutive activation of these tyrosine kinases is crucial to the pathogenesis of certain tumors and myeloproliferative disorders. ^1^^,^^2^  Imatinib is currently considered the first line pharmacotherapy for chronic myeloid leukemia (CML). ^3^^,^^4^  Imatinib is also approved for malignant unresectable or metastatic gastrointestinal stromal tumor (GIST).^   5 ^

Optimization of Imatinib drug dosage regime is important and would affect treatment outcome. To ensure achieving therapeutic threshold trough plasma level (1000 ng/mL) in the majority of patients, a dose of 400 mg per day is advised. ^6^^,^^7^  Imatinib introduced to the Islamic Republic of Iran pharmaceutical market as 100 mg hard gelatin capsule in 2005 (Imatib^®^ by Cipla and Sobhan Oncology). Thereafter concerns regarding treatment adherence were raised because of the need to swallow a high number of capsules each day and over a long period of time to achieve therapeutic dosages. Adherence to prescribed regimens is an increasingly important issue in oncology since exposure to less than required could lead to disease recurrence.^   8 ^ In GIST and some other solid tumor indications, ongoing studies have shown that a dose of 800 mg per day (given twice a day) is superior to a single dose of 400 mg, but has a greater likelihood of non-adherence. ^9^^,^^10^  Two other dosage forms of Imatinib in the market are the 100 mg (Osveh pharmaceutical company, Iran) and 400 mg film coated tablet which was developed and will be introduced to the market in near future. Since the tablets are much smaller and have lower cost than the capsules, patients would have the convenience of a once daily, single, easy-to-swallow tablets of Imatinib. Pharmacokinetic studies have indicated that Imatinib is rapidly absorbed after oral administration, with C_max_ achieved within 2–4 hours. ^11^^,^^12^  Mean absolute bioavailability is 98%, and the elimination half-life of Imatinib and its major metabolite (N-desmethyl Imatinib) are approximately 18 and 40 h, respectively and also the pharmacokinetics of Imatinib is similar in CML and GIST patients. ^13^^-^^15^  In the present study, the bioequivalence, clinical tolerability, and safety of the two tablet formulations (100 and 400 mg tablets) were compared with 100 mg conventional capsules.

## SUBJECTS AND METHODS

**Subjects**

 A group of 36 Iranian (adult male and female) CML patients in chronic phase between 18 and 65 years of age were enrolled and initiated with the standard dose of 400 mg per day as mono therapy. The diagnosis and treatment plan was based on CML guideline of NCCN (Version 4. 2013). The sample size was determined as stated in United State pharmacopeia and thereby the lower limit of subjects for bio equivalency study is 12 individuals in each of groups to differentiate the characteristic and possible differences of formulations. The subjects underwent a medical screening that included medical history, vital signs, and physical and laboratory examinations. Testing was also done for HIV, hepatitis B and C, pregnancy, and alcohol or drug abuse. The other eligibility criteria included renal, hepatic and cardiac function and performance status. The consumer of drugs that have possible metabolism interaction with Imatinib or with a history of smoking was also excluded. In case of severe adverse drug events (in accordance with the Common Toxicity Criteria of the National Cancer Institute), Imatinib dose must be reduced and in such situation patient removed from study. The subjects who missed or discontinued the study prematurely were replaced.

**Study design and analysis**

This was an open label, randomized study in which subjects satisfying the inclusion criteria took either a single dose of 400 mg Imatinib as “four 100 mg hard gelatin capsules (Imatib^®^)”, as “single 400 mg film coated tablet”, or as “four 100 mg film coated tablets (Imatinib-Osveh)”. Subjects were randomly allocated to one of the groups. There were three periods, consisting of a [1] baseline evaluation, [2] the drug administration and [3] a 48 hour post dose pharmacokinetic sampling, and an observation phase. After the sampling period, the patients continued the treatment and followed by a safety phase. The study drug was administered following an overnight fast of at least 10 h for the first dose with a full glass of water, and the subjects continued to fast for at least four hour after dosing.

**Blood sampling**

Several blood samples were obtained from each subject. Samples were collected at time points of before dosing and 0.5, 1, 2, 4, 8, 12, 24 and 48 hour after dosing. Peripheral whole blood (10 mL) was drawn from each subject in EDTA containing tubes, inverted several times, and centrifuged within 30 min at 5000 g for 10 min to obtain the plasma fraction which was transferred into propylene tubes and stored at -20 °C until drug concentration analysis. Samples with unexpected values were not included in the study. 

**Analytical Method**

A validated and sensitive ultra high performance liquid chromatography (UHPLC) method coupled with ultra violet (UV) detection was used to measure plasma Imatinib and N-desmethylate Imatinib concentrations.^        16 ^ The method was validated according to the US Food and Drug Administration (FDA) guidelines for bioanalytical method validation.^   17 ^ The extract plasma was diluted and subjected to a solid-phase extraction. After matrix components elimination, Imatinib was eluted with methanol and the resulting was evaporated and reconstituted with methanol and was injected into the UHPLC system. Imatinib was analyzed using a gradient elution program with solvent mixture constituted of methanol, water, and ammonium acetate. Imatinib was detected by UV detector at 261 nm wavelength. Validation of the analytical method was performed in terms of the validation parameters. All chemicals were of analytic or pharmaceutical grade and used as received. The lower limit of quantification was 50 ng/mL for both Imatinib and N-desmethylate Imatinib. The intraday accuracy (% nominal) was 97.8-107 and 95.6 - 104.5 for the metabolite. The inter day accuracy was between 101-104 % and 98-105% for Imatinib and the metabolite, respectively. The precision (SD for replicate analysis) was from 2.3-9.4 % for Imatinib and 3.1-10.0 % for the metabolite.

**Protocol registration**

Study protocol was approved by the ethics committee of Kerman University of Medical Sciences and conducted in accordance with the Declaration of Helsinki and the FDA guidelines for Good Clinical Practice. It also registered in Protocol Registration System at ClinicalTrials.gov and in Iranian Registry of Clinical Trials (IRCT) by NCT02146846 and 2014050612206N1, respectively. Written informed consent according to institutional regulation was obtained from the participants prior to study entry, (Number of ethic committee: K/92/672)

**Pharmacokinetic analysis**

Pharmacokinetic parameters were determined using noncompartmental method using the Monolix^®^ 4.1.1 software (Lixoft, France). The pharmacokinetic parameters determined included C_max_ and t_max_, which are the maximum concentration observed after dosing and the time at which C_max_ occurred, respectively. In addition, areas under the concentration curves (AUC) were also calculated from time zero to t_max _and 48 h, as indicated. AUC_ (0–48 h)_ were calculated by the linear/log trapezoid method and AUC_ (0–∞)_ was calculated as AUC_ (0–t)_ + C_t_/k_t_ where C_t_ was the concentration at time t, the last measuring sampling time point, and k_t_ was the terminal elimination constant. The elimination half-life (t_1/2_) was determined as 0.693/k. 

**Statistical methods**

The analyses for AUC_ (0–∞)_, AUC_ (0–48 h)_, and C_max_ were regression fits using a linear model for the log-transformed pharmacokinetic parameters. The 90% confidence limits for the difference between least squares means on the logarithmic scale were calculated using Dunnett’s test. The antilogarithm gives the 90% simultaneous confidence limits for the ratio of the two least squares means on the untransformed scale. 

The bioequivalence criterion for the three dosage forms could be considered met if the 90% confidence interval around the ratio of pharmacokinetic parameters, AUC, and C_max_ was entirely contained in the interval (0.8, 1.25). Dunnett’s method was used to calculate 90% confidence limits to ascertain that the overall type I error for comparisons of test formulation versus the reference was equal to 10%. This approach was needed to test whether each of the two strengths of the tablets was bioequivalent to the 100 mg capsule.

**Tolerability assessment**

Toxicities encountered were graded as per the National Cancer Institute’s Common Terminology Criteria for Adverse Events (CTCAE) V.4 (Published: June 14, 2010 By U.S. department of health and human services, National Institutes of Health, National Cancer Institute). Data on adverse events were obtained by patient interview and spontaneous reporting. All the adverse events were recorded.

## Results

**Pharmacokinetic analysis**

 The designed protocol required the enrolment of at least 36 subjects and the removed subjects were subsequently replaced by another one patient. The background and demographic characteristics of the patients who entered the study are summarized in Table 1. The gender of patients in the each groups were equally distributed. The patients’ mean age and body weight was 46 years and 70 kg. Body weight and age distribution of patient was normal and similar in all groups. All other demographic characteristics of patients were checked for normal distribution to prevent any bias.

**Figure 1:**
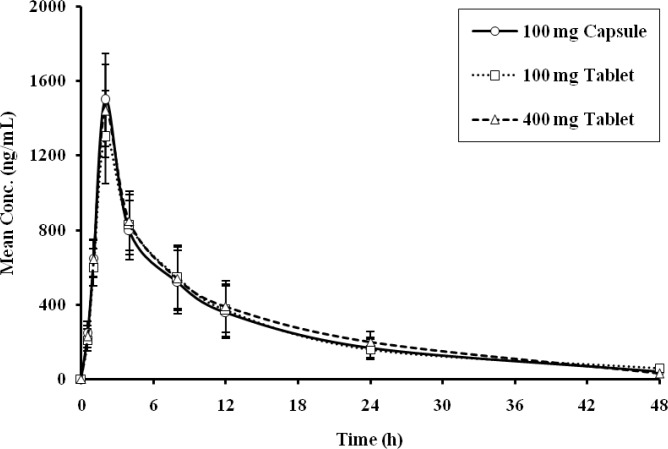
Mean plasma concentrations of Imatinib after administration of different formulation of Imatinib during sampling period

**Figure 2:**
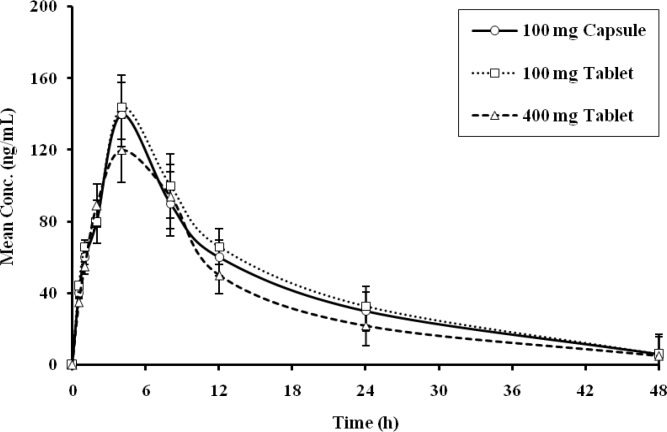
Mean plasma concentrations of Imatinib metabolite after administration of different formulation of Imatinib during sampling period

Figures 1 and 2 represent the mean plasma concentration-time profile of Imatinib and N-desmethylate Imatinib following oral administration of Imatinib either as capsules (4×100 mg) or tablets (4×100 mg or 1×400 mg). These data were used to determine the comparative pharmacokinetic parameters of Imatinib (Table 2) and N-desmethylate Imatinib (Table 3) following administration of the three formulations. The presented data are mean ± SD. Absorption of Imatinib was rapid and unaffected by the formulations. Maximum absorption (C_max_) was found to be comparable between formulations and was achieved in approximately 2 hours for capsules and tablets. Comparable values were also obtained for the AUC measurements of Imatinib at different time points for all three formulations.

**Table 1:** Demographic information of patients evaluated in the study

Demographic characterizations	
Population/Phase	Ph+ Leukemia/Chronic
Number of patients	36
Gender (M/F)	40/34 (54/46 %)
Age (Year)	43 ± 10 (19-63, Median = 44)
BW (Kg)	69.2 ± 8.2 (40.6-89.9, Median = 70.2)
Height (cm)	174 ± 12 (158-190, Median = 171)
BSA (m^2^)	1.71 ± 0.14 (1.29-2.01, Median = 1.69)
Dosage WBC (Cells/mm^3^**)** Platelet (Cells/mm^3^**)**	400 mg per day 9300–169000 (Median = 27700) 100000–1800000 (Median = 420000)

The coefficient of variation for C_max_ and AUCs showed considerable inter subject variability.

The mean plasma concentration-time profile of N-desmethylate Imatinib (Figure 2) demonstrated similar kinetics for both the appearance of Imatinib in plasma after oral administration and breakdown resulting in the metabolite. The AUC of N-desmethylate Imatinib in plasma was found to be about 20% of parent compound for both capsule and tablet formulations, and the t_1/2 _of N-desmethylate Imatinib was double that of the parent compound. The pharmacokinetic values (C_max_, t_max_, and AUC) for N-desmethylate Imatinib were similar for different formulations of Imatinib. The test/reference ratios for AUC _(0–inf)_, AUC_(0–48 h)_, and C_max_ were 0.99, 0.99 and 1.02 for 4 × 100 mg tablets versus 4 × 100 mg capsules, and 0.96, 0.96 

**Table 2:** Imatinib pharmacokinetic parameters following oral administration of Imatinib capsule or tablets (The data presented as Mean ± SD)

**Parameter**	**Capsule** **(4 × 100 mg)**	**Tablet** **(4 × 100 mg)**	**Tablet** **(1 × 400 mg)**
T_max_ (h)	2.0 (1.5 - 3.5)	2.1 (1.2-3.8)	2.0 (0.9-3.9)
C_max_ (ng/mL)	1548 ± 252	1605 ± 209	1622 ± 233
t_1/2_ (h)	15.7 ± 2.2	15.8 ± 2.8	15.6 ± 2.2
AUC_(0-2)_ (ng × h/mL)	2324 ± 922	2199 ± 988	2019 ± 983
Auc_(0-48)_ (ng × h/mL)	19834 ± 8215	19003 ± 7563	18452 ± 8235
AUC_(0-∞)_ (ng × h/mL)	27011 ±11562	25811 ± 10547	25699 ± 11253
Volume of distribution (L)	382 ± 112	384 ± 105	391 ± 99
Clearance (L/h)	17.2 ± 3.7	17.1 ± 4.2	17.8 ± 5.4

**Table 3:** Imatinib metabolite (N-desmethylate) pharmacokinetic parameters following oral administration of Imatinib capsule or tablets (The data presented as Mean ± SD)

**Parameter**	**Capsule** ** (4 × 100 mg)**	**Tablet ** **(4 × 100 mg)**	**Tablet ** **(1 × 400 mg)**
T_max_ (h)	2.5 (1.5 – 6.4)	2.8 (1.3 – 7.1)	2.6 (1.9 – 6.5)
C_max_ (ng/mL)	201 ± 72	199 ± 77	189 ± 62
t_1/2_ (h)	36.2 ± 6.1	38.2 ± 9.3	41.0 ± 9.6
AUC _(0-2)_ (ng × h/mL)	277 ± 103	281 ± 126	244 ± 98
Auc _(0-48)_ (ng × h/mL)	2445 ± 1022	2411 ± 902	2321 ± 812
AUC _(0-∞)_ (ng × h/mL)	5243 ± 2156	5303 ± 2109	5142 ± 2003

and 0.99 for 1 × 400 mg tablet versus 4 × 100 mg capsules (Table 4). Dunnett’s adjusted confidence limits for each of these parameters were in the interval (0.80, 1.25), as were unadjusted confidence limits. The test/reference ratios of pharmacokinetic parameters obtained indicated the bioequivalence of the three different formulations of Imatinib tested.

**Safety and tolerability**

During the study 77 adverse events were reported in 19 of the 36 subjects; some subjects experienced more than one adverse event and from 77 adverse events, 66 were thought to be related to Imatinib and were either mild (grade 1, n = 46) or moderate (grade 2, n = 20) in severity. 

The adverse events were categorized according to System Organ Class (SOC), the highest level of the MedDRA hierarchy, is identified by anatomical or physiological system, etiology, or purpose. As the study duration was limited, the latent adverse events such as renal, hematologic, hepatic, endocrine and metabolic disorder were not included. The reported adverse events were mostly from gastrointestinal, CNS, neuromuscular, cardiovascular and skin disorder.

The frequency and intensity of adverse events was comparable across all formulations of Imatinib (Table 5). The adverse events’ frequency and severity between studies groups was compared by analysis of variances and using the P_value_ (more than 0.05) which showed that the differences between groups were not meaningful. Clinical laboratory evaluations (biochemistry, hematology and urinalysis) and monitoring for vital signs revealed no abnormalities that could be clinically significant or related to the use of Imatinib during the study period.

**Table 4:** Statistical comparisons of the ratios (test/reference) of the geometric means of pharmacokinetic parameters

**Parameter**	**Treatment**	**Geometric ** **mean**	**Ratio of ** **geometric mean**	**90% Dunnett ** **adjusted CI for ratio**
AUC _(0-∞)_ (ng × h/mL)	Capsule (4 × 100 mg)	24321	Reference	
	Tablet (4 × 100 mg)	24176	0.99	(0.91, 1.04)
	Tablet (1 × 400 mg)	23357	0.96	(0.89, 1.01)
Auc _(0-48)_ (ng × h/mL)	Capsule (4 × 100 mg)	24218	Reference	
	Tablet (4 × 100 mg)	24003	0.99	(0.91, 1.04)
	Tablet (1 × 400 mg)	23288	0.96	(0.89, 1.01)
C_max_ (ng/mL)	Capsule (4 × 100 mg)	1512	Reference	
	Tablet (4 × 100 mg)	1557	1.02	(0.89, 1.05)
	Tablet (1 × 400 mg)	1498	0.99	(0.88, 1.04)

**Table 5:** Frequency and distribution of adverse events according to grade (all were grades 1 or 2) with a possible or suspected relation to Imatinib during the study period, by formulation category

**Adverse events**		**Frequency of adverse events with suspected relation to Imatinib**					
		**Capsule** **(4 × 100 mg)**	**Tablet ** **(4 × 100 mg)**	**Tablet ** **(1 × 400 mg)**	**Total ** **(%)**	**P**_value_	**Frequency** **(%)**
**Gastrointestinal**	Nausea	3	3	4	10 (27.8)	>0.1	15.2
	Diarrhea	0	1	1	2 (5.5)	>0.1	3.0
	Abdominal pain	2	4	6	12 (33.4)	>0.1	18.2
**CNS**	Headache	3	2	4	9 (25)	>0.1	13.6
**General**	Fatigue	4	2	5	11 (30.5)	>0.1	16.7
	Edema (limbs)	4	2	2	8 (22.3)	>0.1	12.1
**Musculoskeletal**	Myalgia	2	3	3	8 (22.3)	>0.1	12.1
**Skin**	Rash	2	1	3	6 (16.7)	>0.1	9.1

## Discussion

 The current study demonstrated comparable bioavailability of Imatinib 4 × 100 mg capsules with 4 × 100 mg tablets and 1 × 400 mg tablet. The pharmacokinetic values (C_max_, t_max_, and AUC) for N-desmethylate Imatinib were also found to be nearly identical for both the test and reference dosage formulation of Imatinib. The similar pharmacokinetic property of the metabolite further supports the bioequivalence of the tablet product. Previous laboratory tests have indicated that the tablet formulations of Imatinib can dissolve as easily and over the same pH range (1.0–6.8) as the capsule. This study indicated rapid absorption of the 400 mg tablet (median t_max_ 2 hours) after oral administration that was comparable with that of the capsule. Taken together, these results demonstrate that Imatinib is as highly soluble and as rapidly absorbed in tablet formulation as in capsule form. The coefficient for variation for C_max_ and AUCs showed considerable inter subject variability. Although, the causes was not clear, it may be attributed to inter subject differences in plasma proteins binding to the parent compound or to variations in CYP3A4, the major cytochrome isoenzyme involved in the metabolism of Imatinib. Variability in CYP3A activity between individuals is large, ^18^^,^^19^  and may in part have contributed to the large inter subject variability.

To reach the goal of the study, patients with the similar demographic characteristics was selected, so the comparison would be more realistic and it could be possible to differentiate between the formulations and their differences would be arise.

In this study, the patients did not take any other medication and dietary supplements except Imatinib during study period and all the reported adverse events may attribute to the Imatinib.

Adverse effects were closely monitored and found to have a similar distribution among the three groups. None of these effects showed clinical significance. The types of adverse events observed in this study are consistent with those reported in other studies.^20^^-^^23^ The data presented in this study clearly demonstrate the safety and tolerability of the 400 mg formulation of Imatinib. 

In the present study there are several limitations such as small sample size, short duration of study and not letting cross over happen which are due to ethical limitations and characteristics of treatment regimen. It is not possible to prevent the patients from taking Imatinib for two weeks (necessary washout period between cross over study) or changing the drug formulations. Designing a study with patients in two groups with similar demographic characteristics could make it possible to discover the possible differences between formulations.

## CONCLUSION

 The advantages of the 400 mg tablets include (1) convenience for patients who, in most cases, will need to take only one tablet per day (instead of four 100 mg capsules), (2) the potential to sustain adherence over the long run and (3) lower costs of treatment. The 100 mg tablet also offers dosing flexibility for the pediatric patient population, for which the recommended dose is 260–340 mg/m^2^ per day. In conclusion, the bioavailability, safety, and tolerability of the 100 mg and 400 mg film coated tablet formulations of Imatinib are comparable to those of the 100 mg hard gelatin capsule. The tablets can be safely prescribed to replace the capsules in the treatment of CML and GIST.
